# Optimizing electrotactile stimulation intensity for responses under cognitive load

**DOI:** 10.1186/s12984-026-01883-1

**Published:** 2026-02-13

**Authors:** Felix Jarto, Elaine Corbett, Sigrid Dupan

**Affiliations:** 1 School of Electrical and Electronic Engineering, University College Dublin, Dublin, Ireland; 2 School of Mechanical and Materials Engineering, University College Dublin, Dublin, Ireland; 3 Centre for Biomedical Enginering, University College Dublin, Dublin, Ireland

## Abstract

**Background:**

Prosthetic users mainly rely on their vision for feedback during control of the device in absence of the tactile and proprioceptive modalities. Overreliance on vision increases cognitive load during prosthesis use, resulting in reduced control performance. Provision of additional feedback through other modalities could reduce this overreliance on vision and improve closed-loop prosthetic control. Fast and accurate recognition of feedback is particularly important when we consider that both delays and inaccuracies can destabilize closed-loop control environments. Transcutaneous electrotactile stimulation is a promising technology with advantages including non-invasiveness and simplicity. It is not yet clear, however, which parameters influence the speed and accuracy of response to electrotactile stimuli. In this study, we set out to investigate how we can influence response characteristics to an instantaneous change in stimulation intensity by manipulating both stimulus-related and environmental variables.

**Methods:**

20 participants completed a randomized reaction time test to both visual and electrotactile stimulation. Participants were asked to respond to incoming stimuli with a button press on a controller as fast as possible, without discriminating between modality of stimulation. In a second experiment, 20 participants completed an intensity discrimination task for electrotactile stimulation. Participants had to prioritize either speed or accuracy during specific blocks, while cognitive load, magnitude and direction of stimulus shift were manipulated.

**Results:**

The results of the reaction time experiment confirmed faster average response times for electrotactile stimuli compared to visual stimuli by a median of ~ 50 ms (*p* < 0.01). In the intensity discrimination experiment, increased shift intensities led to increased response accuracies and faster response times (*p* < 0.001 in both cases). The presence of cognitive load slowed average response times (*p* < 0.01), but did not affect response accuracies (*p* = 0.42).

**Conclusions:**

The results of the intensity discrimination experiment imply that the magnitude of stimulus shift needs to be provided via steps multiple times larger than the just noticeable difference to ensure fast and accurate responses to electrotactile stimulation. Moreover, results from the reaction time experiment confirmed faster average response times for electrotactile stimuli over visual stimuli. We argue that providing electrotactile stimulation using bigger steps in perceived intensity could result in the supplementary feedback making closed-loop control of prosthetic devices more reliable.

## Introduction

In the last two decades upper limb prosthetics have seen many technological improvements, yet rejection rates continue to remain at levels as high as 44% [1]. Previous studies have shown that major reasons behind device abandonment include, among others, unreliable control and lack of sensory feedback [2, 3]. The topic of sensory feedback - both invasive and non-invasive - has been heavily investigated in recent years: many studies have found promising results regarding enhanced functionality [4], promotion of embodiment [5], decrease in cognitive load [6] and alleviation of phantom limb pain [7]. However, it is difficult to draw conclusions, as currently there is no gold standard for sensory feedback and many different feedback strategies are employed in the aforementioned studies. Moreover, quality-of-life improvements outside of the lab have not been established yet. There is a lack of data collected in home environments [8–10], and those that collected data at home reported significant variabilities in the performances of their subjects [11]. Sensinger and Dosen hypothesize that mixed outcomes and, by extension, the lack of sensory feedback in commercially available prostheses are a result of insufficient understanding about the role and mechanisms of feedback in prosthetic control [12]. Thus, to improve sensory feedback paradigms for prosthetics, it is crucial to investigate sensory feedback through the lens of sensorimotor control.

Despite changes to both our body and the environment over time, the motor system produces accurate goal-directed movements. However, when studying movement in more detail, it is clear that realized movements do not always align with our intentions. One contributing factor to this misalignment is inherent noise in the sensory and motor systems, mainly related to synaptic activity [13]. Another important factor is that movement is based on internal models representing our body and the environment [14]. When changes happen, e.g. when our muscles are fatigued, misalignment will occur. When the executed movement doesn’t align with our intentions, sensory feedback allows us to perceive the difference, make small corrections on the fly and update our internal models [15]. In any kind of closed-loop control environment, sensory feedback delays have to be minimized to improve control stability [16]: if the feedback arrives too late, it might prove irrelevant to the momentary movement and the ability to update our internal models will be lost due to the inability to establish an accurate causal relationship between performed action and perceived feedback.

The speed of feedback is particularly important when we consider the mechanisms of the decision making process inside the brain [17, 18]. When a decision is required, the brain gathers so-called evidence in the form of incoming sensory information [19]. In the case of movement, we mainly rely on the modalities of vision, touch and proprioception to provide us with information regarding internal and external states to ensure fluid and accurate movement [12]. These sources are integrated by weighting them based on the certainty of the information; enough evidence has been gathered when a certain threshold is passed, resulting in a decision [20]. The speed and accuracy of our decision depends on the level of this decision-threshold: if the threshold is low, less evidence is needed to make a decision, which speeds up the process while sacrificing accuracy. On the other hand, a higher threshold requires more evidence, which raises the likelihood of a correct answer, but makes the process slower. Due to the decision making process needing time to accumulate evidence necessary to make a judgment, the sensorimotor system is faced with a dilemma: it can make a fast decision which compromises the response accuracy or it can wait for more evidence, compromising the reaction time. In order to minimize the effect of this tradeoff on real-time control, it is not enough to provide sensory feedback through a fast modality, but the feedback also needs to include all the necessary information within a short timescale.

The speed of processing and the impact of the incoming information are different across modalities. These differences are reflected in reaction times, and are dependent on many factors, such as attention, conduction time and complexity of stimuli [21]. However, generally, reactions to tactile stimuli are faster than to visual stimuli [22–24]. When integrating information from different modalities, how much information is taken from a specific modality is related to the weighting given during sensory integration. The weighting depends on the certainty of the signal, where a modality with high certainty will be weighted higher than those with low certainty [25]. As a result, adding feedback is not always going to improve performance: if the supplementary feedback carries a high amount of uncertainty, sensory integration will be inefficient and the impact in control will be negligible. Thus, to be effective, supplementary feedback has to be fast and distinguishable in order to be assigned higher weighting.

In the case of individuals with limb-difference, the tactile and proprioceptive modality are almost completely lost, resulting in difficulties for people to localize their limbs in space, as well as losing the ability to accurately sense their prosthetic limbs interacting with the environment. To compensate, people rely on vision and, in some cases, incidental feedback such as auditory cues [26]. As visual stimuli are generally processed more slowly than tactile ones [22], this potentially introduces a significant control delay to the system. This delay is further increased in the case of myoelectric devices due to how EMG signals are usually smoothed via windowing methods [27], which are dependent on collecting data over a certain amount of time before an output can be generated. Furthermore, the overreliance on vision imposes a heavy cognitive load during prosthetic control, which hinders the use of the device [28]. In order to alleviate the load on the visual system and improve feedback control, supplementary feedback through other modalities should be provided.

If we want feedback that integrates well into the sensorimotor loop, we need to provide it in a way that is fast, easily recognizable and distinguishable. In this study, we investigated how transcutaneous electrotactile stimulation can fulfill these requirements. Electrotactile stimulation is an attractive choice due its non-invasive and energy efficient nature [29]. Furthermore, it allows for direct cutaneous stimulation with high temporal precision. Given our focus on response speed and discrimination accuracy, we employed electrotactile stimulation to enable fine-grained control of stimulus timing and intensity. While vibrotactile stimulation is an equally attractive modality to provide sensory feedback, the motor inertia of the vibrator could cause delays in timing of stimulation [30, 31]. Electrotactile stimulation has also been used before to close the loop during prosthetic control, either by conveying information regarding sense of touch useful to determine grasping force [32, 33] or proprioception which helps keep track of joint angles [34]. Because of the importance of speed in sensorimotor control, we first aimed to confirm that electrotactile stimulation registers more quickly than visual stimulation. In a second experiment, we investigated how different stimulation- and environment-related parameters can influence the speed-accuracy tradeoff (SAT), with the goal of identifying parameters that lead to both fast and accurate decision-making. Both of these experiments included simple motor tasks in order to better separate sources of delay and inaccuracy in the sensory system without the interference of a more complex motor task, such as myoelectric control. We hypothesize that (1) electrotactile stimulation will show faster reaction times compared to visual stimulation, (2) improvements in accuracy and response times will be seen for larger shift intensities, (3) presence of cognitive load will negatively affect response times and response accuracies and (4) instructions to focus on speed or accuracy will favorably affect the focused outcome metric, possibly to the detriment of the un-focused metric.

## Methods

Participants provided written informed consent before the start of the experiments, agreeing to take part in the study and their data, once anonymized, to be used and shared. The study was approved by the UCD Human Research Ethics Committee–Sciences (LS-22–46-Dupan).

### Experiment 1: reaction time

#### Experimental protocol and design

Twenty participants (9 male, 11 female, with mean age ± SD = 29 ± 5 years) without limb difference took part in the experiment. Participants sat in front of a monitor and provided their responses using a game controller (CH Products Flight Stick Pro 200–503). A pair of electrodes (Axelgaard PALS 5 × 5 flex electrodes, USA) were positioned on the anterior side of the non-dominant forearm of the participants, slightly above the wrist and along the flexor carpi radialis. Stimulation was delivered to the contralateral forearm to eliminate potential interference from involuntary motor activity elicited by the stimulation. This design allowed us to assess fundamental aspects of stimulus perception and intensity discrimination independently of prosthetic control dynamics. Electrotactile stimulation was provided using an analog stimulus isolator (A-M Systems Model 2200, USA) which was controlled by an Arduino Uno microcontroller connected to the experimental PC running a Python script. Stimulation was provided as monophasic square waves with amplitude and frequency fixed at 3 mA and 50 Hz respectively. The use of 50 Hz takes the refractory period of sensory fibres into account by not exceeding 125 Hz [35]. The frequency was further narrowed down as intensity discrimination for electrotactile stimulation was shown to be better at lower frequencies (12.5–87.5 Hz) than higher ones (50–200 Hz) [36]. For amplitude, the 2–4 mA range has been reported to be clearly perceptible while not causing significant discomfort by previous studies employing pulse-width modulation in electrotactile stimulation [33, 37]. The perceived stimulus intensity was modulated by increasing and decreasing the pulse width of stimulation trains.

**Fig. 1:**
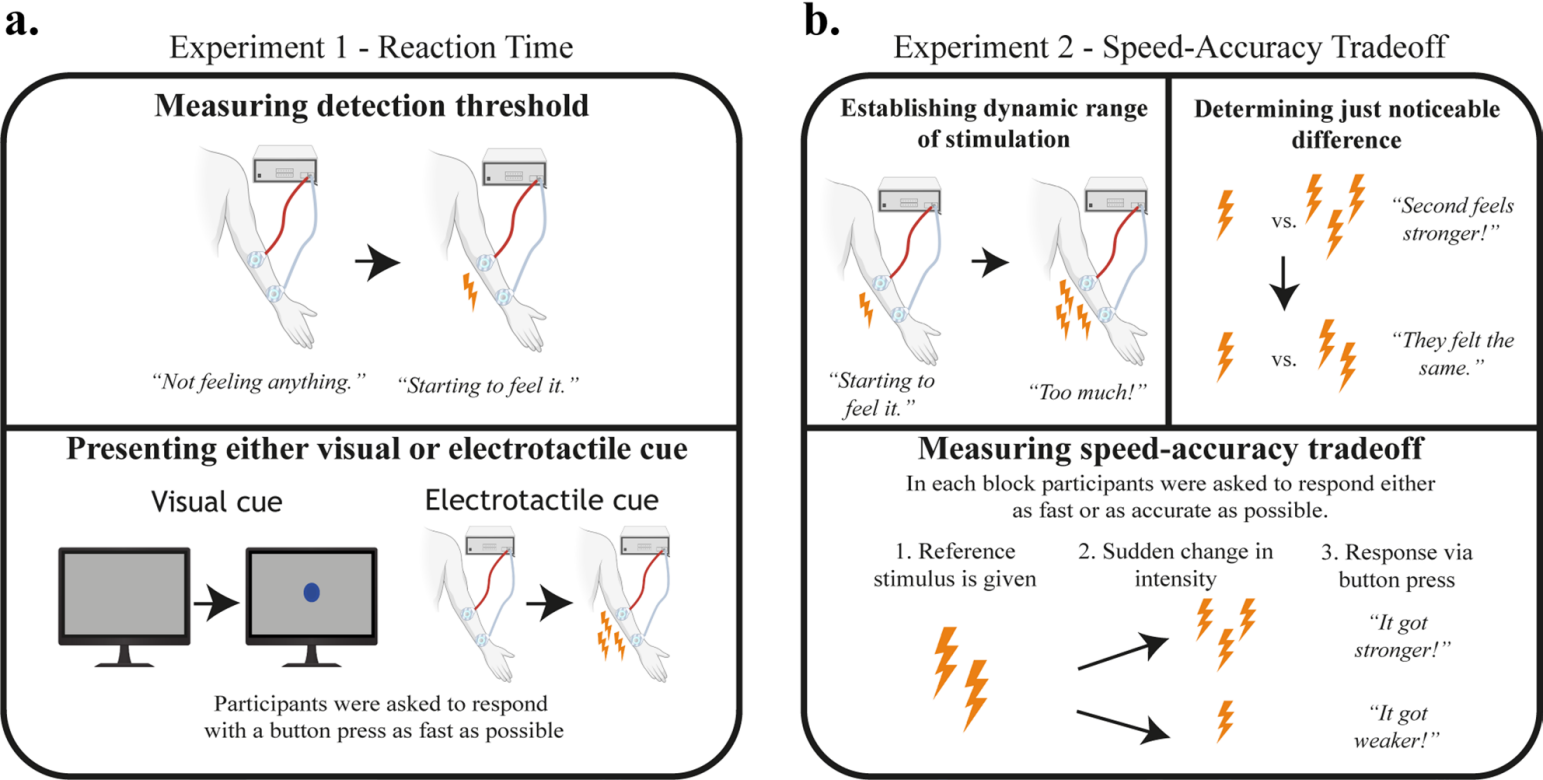
Experimental methods. **a** Reaction time experiment methodology. Subject is randomly presented with either a visual or an electrotactile stimulus. They have to respond with a button press as fast as possible. No discrimination between types of stimuli have taken place. **b** Speed-Accuracy Tradeoff experiment methodology. At the start, the dynamic range (DR) is established via measuring the detection threshold (DT) and pain threshold (PT). Then participants are presented with two consecutive stimuli with a small delay inbetween to establish the just noticeable difference (JND). Finally, participants indicated if the first or second stimulus felt stronger after an instantaneous fall or rise in intensity

The experiment consisted of 2 parts, which are represented in Fig. 1a. First, the detection threshold (DT) was determined by increasing the stimulation pulse width from 50 µs. The initial pulse width value was chosen to be an intensity most likely unperceivable to participants [37]. Pulse width was increased in increments of 50 µs during thresholding. Participants were asked to press a button on the controller when they felt the stimulation, which resulted in the pulse width dropping by 100 µs. The DT was defined as the intensity at which the participant indicated a perception event 3 consecutive times.

During the reaction time task participants were asked to press the button on the game controller as fast as possible after perceiving a stimulus. Stimuli were either a visual stimulus (a circular dot appearing on the monitor) or an electrotactile stimulus. The intensity of the electrotactile stimuli were set to twice the value of the DT. Initial pilot studies (*n* = 3) confirmed that this level of stimulation was easily perceptible without causing any uncomfortable or painful sensations. The experiment consisted of 2 blocks with 40 trials each. Each block consisted of an equal number of visual and electrotactile stimuli. The order of the visual and electrotactile stimuli were randomized on the level of individual trials.

The start of each trial was indicated by a visual cue appearing on the screen (‘start cue’). The start cue remained on the screen until either the visual stimulus appeared in its place or - in the case of electrotactile stimulus - the end of the trial. Participants were informed that they could receive either stimulation while the start cue is present on the screen. Unknown to the participants, the foreperiod (time between start cue and stimulus onset [38]) was varied between 2–4 s to avoid the participants predicting the time of stimulus onset. End of trial was denoted by a different visual cue (‘end cue’). The end cue remained on screen for the entire duration of the 3 s inter-trial interval (ITI). ITI’s of 3 s have previously been shown to have a favorable effect on reaction times [39, 40]. A between-block break of ~ 3 min was given to participants after finishing block 1. The stimuli in both conditions were deemed to be easily noticeable in pilot trials, thus a timeout condition was not established.

#### Data analysis

The main outcome variable used to determine the difference in processing speed between visual and electrotactile stimulation was reaction time. For each feedback modality, average and standard deviation of reaction times were determined for each participant. These values were used for subsequent analysis. Normality was tested using the Shapiro–Wilk method which confirmed normal distribution of the averages, but not for the standard deviations. Therefore, we used the Wilcoxon signed rank test for all comparisons. Furthermore, we fit an exponentially modified gaussian distribution to the pooled reaction times from all participants. This was done in order to confirm that both sets of reaction times conform to established distribution patterns [41, 42]. For the fitting of the exponentially modified gaussian, we used the *Exponentially modified Gaussian (ex-Gaussian) distributions toolbox* available through MATLAB File Exchange [43].

### Experiment 2: Speed-Accuracy Tradeoff (SAT)

#### Experimental protocol and design

Twenty participants (11 male, 9 female with mean age ± SD = 28 ± 5 years) without limb difference took part in the experiment. Participants sat in front of a monitor and provided their responses using a numeric keypad. Similarly to the first experiment, the electrotactile stimulation was provided using an analog stimulus isolator (A-M Systems Model 2200). The stimulus isolator was controlled using an NI board (NI USB-6211) connected to the experimental PC running a Python script. Stimulator electrodes were placed above the flexor carpi radialis of the non-dominant forearm of the participants. Stimulation was provided as biphasic square waves, with frequency and amplitude fixed at 50 Hz and 5 mA respectively. Due to the higher amplitude of stimulation enabled by the NI board, the stimulation electrodes were placed along the upper part of the flexor carpi radialis; this elicited a much more defined sensation compared to the 3 mA stimulation provided by the Arduino board. Perceived intensity of stimuli was modulated by increasing and decreasing the pulse width.

Figure 1b shows the 3 parts of this experiment. First, we measured the dynamic range (DR) of stimulation via establishing the detection threshold (DT) and pain threshold (PT). The DT was determined the same way as in the reaction time experiment. Following that, the pulse width was increased to a point where the participant reported a feeling of discomfort or pain. Similarly to the DT, this was done in small increments of 50 µs which minimized the risk of administering a substantially painful stimulation after a relatively comfortable level. No participants indicated any painful sensations verbally when asked about the intensity of the PT, most of them citing discomfort. For the comfort of the participants, this level was only recorded once, and was noted as the pain threshold (PT). The DR was determined as the pulse width range between the DT to the PT.

In the second part, the just noticeable difference (JND) was measured with respect to a stimulus set to 25% of the DR using a staircase procedure. The procedure began with the participant receiving two consecutive stimuli with a 0.5 s pause in between each; one stimulus was set to 25% of the DR (reference stimulus) and the other set to 50% of the DR (compared stimulus). In each of the trials, the order of the reference and compared stimuli were randomized. The participant was instructed to determine the stronger of two consecutive stimuli after receiving both. Every correct response resulted in the pulse width of the compared stimulus dropping by 10 µs and every incorrect response in the pulse width rising by 20 µs. An incorrect response followed by a correct one, or a correct response followed by an incorrect one were defined as reversals. JND was determined as the difference between the reference intensity and the average of the ten intensities where a reversal has taken place. The maximum intensity of stimulation during the experimental protocol was fixed to 25% of the DR + 4 times JND. This maximum intensity was compared to the PT after thresholding: if this level exceeded the PT, the experiment would be halted and the participant were to be excluded. We did not encounter this during the experiment.

During the main SAT task, participants were asked to respond to an instantaneous shift in stimulus intensity via two buttons on a keyboard. Button 1 indicated the first received stimulus felt stronger (downward stimulus shift), while button 2 indicated that the second stimulus felt stronger (upward stimulus shift). The instantaneous shift aimed to replicate the provision of electrotactile feedback during continuous prosthetic control. The reference intensity was set at 25% DR and the magnitude of change in intensity, here denoted as shift intensity, corresponded to 1–4 times JND, in steps of 1xJND. The direction of stimulus shift (rising/falling) was pseudorandomized over trials. The start of the trial was indicated by the onset of the first stimulus, accompanied by the start cue on the screen, and lasted between 2 and 4 s. The onset of the second stimulus coincided with the end of the first, while the visual cue remained the same. The second stimulus continued until the participants responded with a button press indicating the direction of the change or timed out after 3 s. A different visual indication (the same ’end cross’ as in experiment 1) appeared after the button press or timeout denoting the start of the ITI. In case of timeout, the participant’s response was classified as a “missed response”.

The SAT task consisted of 20 blocks in total, containing 24 trials each. In each block, the levels of shift intensity and shift direction were balanced, while their order was randomized. This meant that in every block, participants received 3 upwards and 3 downwards shifts per shift intensity, bringing the number of trials to 24. At the start of each block, participants were instructed to prioritize speed or accuracy in their response via a text on their screen. This condition was alternated between every block, with the first block’s condition being pseudorandomized between participants, and was indicated by the following text on screen: “In this block you must respond as [FAST/ACCURATE] as possible.” Furthermore, an added cognitive load condition was present in half of the blocks. The presence of cognitive load was alternated every two blocks, with the first block’s condition being pseudorandomized between participants. This condition was introduced in order to investigate how the increased cognitive load present during prosthesis use [28] affects the speed and accuracy of recognizing changes in electrotactile stimulation. To impose cognitive load, participants were asked to listen to paragraphs from an audiobook (Dr. Ox’s Experiment by Jules Verne) during the block, and answer multiple choice questions (MCQ) immediately after the conclusion of these blocks [44–46]. Before blocks featuring the cognitive load dual task, participants received an additional message: “In this block you will listen to the audiobook”. The outcome variables of the task were response speed and response accuracy.

#### Data analysis

The main outcome variables used to determine the impact of stimulation parameters in instantaneously shifting electrotactile stimuli were reaction time and response accuracy. The independent variables we considered were: (1) stimulus shift intensity, (2) stimulus shift direction, (3) presence of added cognitive load and (4) explicit instruction regarding focus on speed or accuracy of response.

An ANOVA test was performed to determine which stimulation parameters had an effect and whether interactions were present between them for both reaction times and response accuracies. Only the per-condition average reaction times and response accuracies were considered for each participant. Normality was tested using the Shapiro–Wilk method for all subsets of the data which confirmed normality in 13 of 20 subsets. Even though an ANOVA was used to determine interactions and individual effect, due to the high amount of non-normal subsets, we used the Wilcoxon signed rank test to compare conditions. In the case of shift intensity comparisons, Bonferroni correction was applied to the p-values of Wilcoxon signed rank tests. Post-hoc Wilcoxon signed-rank tests were performed to determine whether cognitive load had an effect on the number of missed responses at all shift intensity levels.

Additionally, we performed post-hoc tests to determine whether short- and long-term sensory habituation to electrotactile stimulation could have influenced our results [47, 48]. We divided all trials into “onset” and “terminal” categories, as well as “early” and “late” categories. All trials featured in the first half of their respective blocks were assigned the “onset” label, while ones featured in the second half were categorized as “terminal” (trial 12 being the cutoff point in all blocks, still labeled “onset”). Similarly, all trials were sorted into either “early” or “late” categories, depending on whether they took place in blocks featured in the first or second half of the experiment (block 10 being the cutoff point). Response times and response accuracies were averaged across participants in all categories and were compared using a Wilcoxon signed-rank test with their respective pairs (“onset” with “terminal” and “early” with “late”).

## Results

### Experiment 1: reaction time

**Fig. 2:**
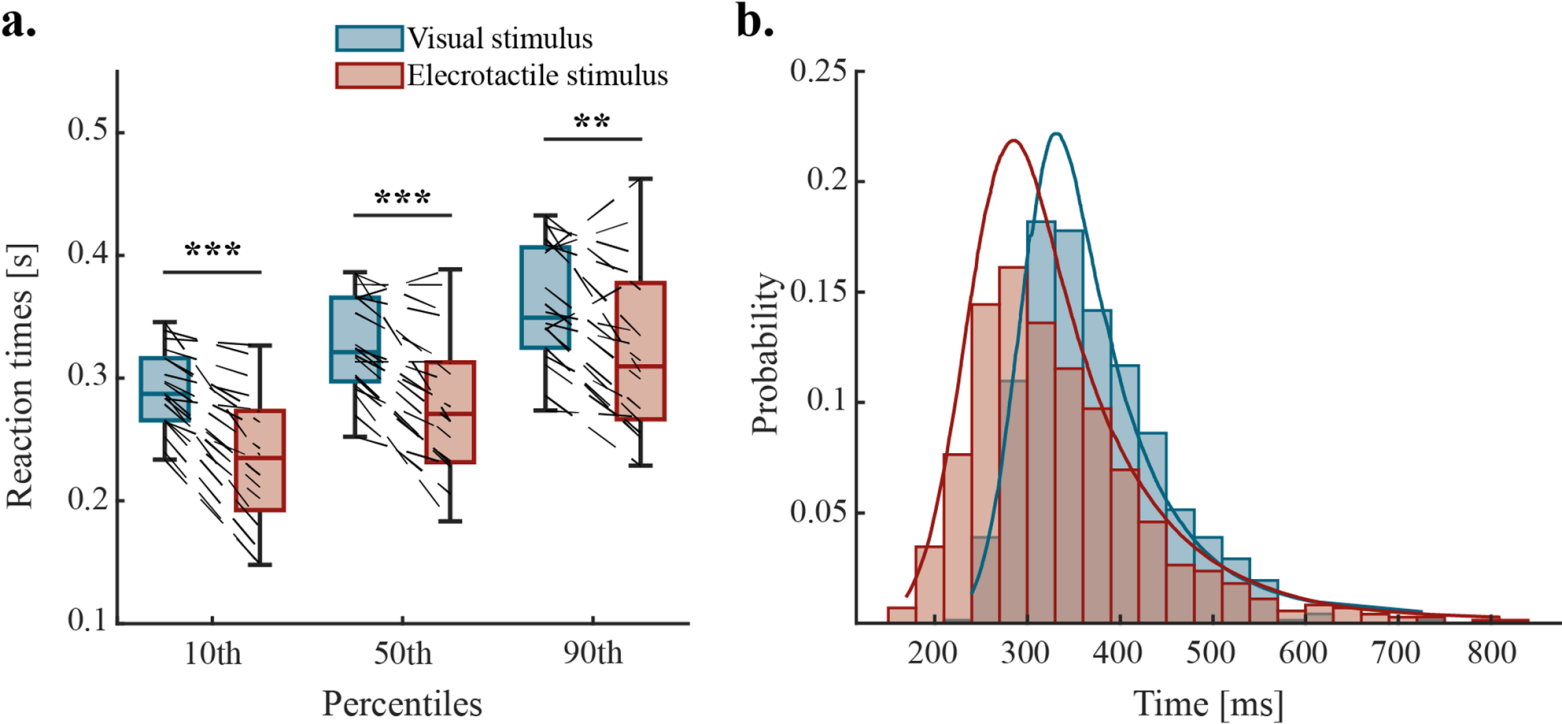
Results of the reaction time experiment. **a** Average reaction times are significantly faster for electrotactile stimulation in the 10th, 50th and 90th percentiles (*p* < 0.001, *p* < 0.001 and *p* < 0.01 respectively). Connected data points signify the percentile averages for individual participants; **b** Distribution of pooled reaction times. Reaction times to both stimuli follows an exponentially modified gaussian distribution

We first tested whether there is a significant difference in reaction times between visual and electrotactile stimulation. Based on existing literature stating that tactile stimulation registers faster than visual stimulation in general [22–24], our tests aimed to confirm whether this is true for electrotactile stimulation as well. A Wilcoxon signed-rank comparison of average reaction times for visual stimuli (Mdn_VIS_=359.74 ms, IQR = 334.82–422.14 ms) and electrotactile stimuli (Mdn_ELE_=329.33 ms, IQR = 281.05–389.32 ms) revealed significantly faster reaction times to electrotactile stimulation (*p* < 0.01). Comparing the medians revealed a 30.41 ms difference between the two modalities, with electrotactile stimulation being faster. Taking the averages of the 10th, 50th and 90th percentiles of each participant’s reaction times showed significantly faster reaction times in all three percentiles (Fig. 2a). This suggests that electrotactile stimulation doesn’t show significantly increased variability compared to visual stimulation while allowing faster reactions on average. Figure 2b shows the pooled reaction times of all participants, with an exponentially modified gaussian distribution fitted to both stimulus modalities [41, 42].

### Experiment 2: SAT

The second experiment investigated whether the SAT can be influenced by modulating stimulus-related (stimulus shift direction and magnitude) and environmental variables (explicit instructions and presence of cognitive load) during shifting electrotactile stimulation. Identifying the parameters that ensure fast and accurate perception of electrotactile stimuli is a prerequisite for implementation of electrotactile feedback in prosthetic control. We began the experiment by determining the DT, PT and JND of the participant (Avg_DT_: 190.25 ± 79.84 µs, Avg_PT_: 612.50 ± 168.49 µs, Avg_JND_: 37.45 ± 16.51 µs). Our thresholding results were largely consistent with previously reported ranges of electrotactile stimulation [37]. While the maximum intensity of stimulation exceeding the PT was an exclusion factor, this did not affect any of the participants. The experiment was aimed to measure reaction times and correct responses to instantaneous shifts in electrotactile stimulation under four different sub-conditions: stimulus shift direction (rising and falling intensity), stimulus shift intensity (1-4xJND value), explicit instruction (focus on speed or accuracy of response) and concurrent cognitive load (dual audiobook task or no additional task).

**Fig. 3:**
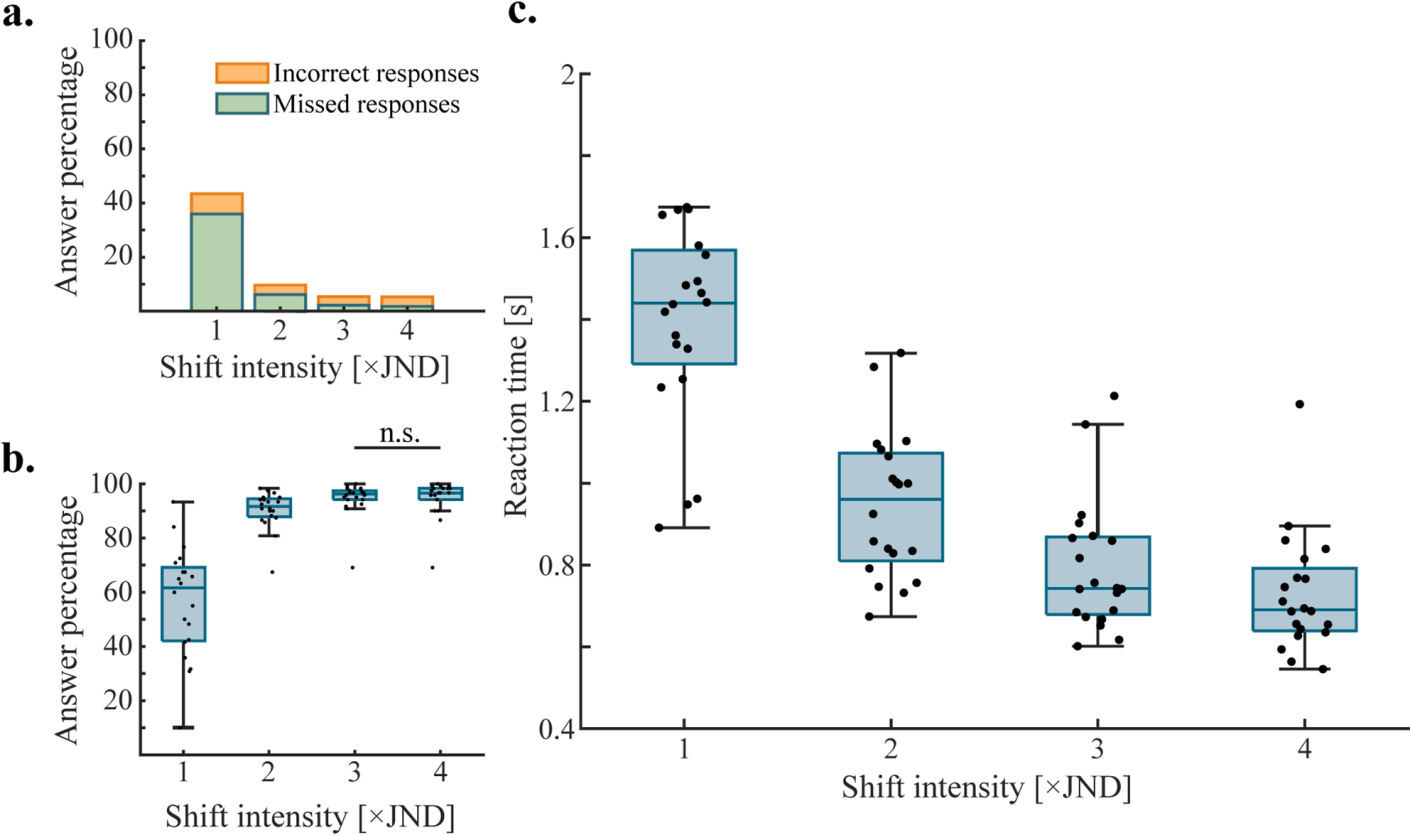
Response accuracies and reaction times as a function of shift intensity; **a** The number of incorrect and missed responses decrease as shift intensity gets larger. **b** Average response accuracy per participant increases with shift intensity. Black dots denote averages of individual participants. Only non-significant differences are shown, all other comparisons are significantly different. **c** Average reaction times per participant decrease with shift intensity; only correct responses were considered in this case. Black dots denote averages of individual participants. All comparisons were significantly different

Four-way ANOVA was performed on both reaction time and response accuracy data in order to determine which variables had the most effect on the outcomes, as well as to find interaction effects between them. The ANOVA revealed that stimulus shift intensity had the strongest effect on both response accuracies and reaction times (*p* < 0.001 in both cases). Significant differences can be seen for all intensities in response accuracies, except for 3x and 4xJND values (*p* < 0.001 for all 1xJND comparisons, *p* < 0.01 for comparison between 2x and 3xJND; Fig. 3b). Pairwise comparisons show significant differences across all intensities for response times (*p* < 0.001 in all cases; Fig. 3c).

In the pooled responses of participants, most response inaccuracies were observed at the lowest shift intensity, the majority of which were missed responses (Fig. 3a): median of average incorrect responses per participant at 1xJND shift intensity was 37.46% (IQR = 32.03% − 56.43%), with a median of 29.01% (IQR = 18.43% − 55.98%) for average missed responses. The number of missed and inaccurate responses decreased significantly as shift intensity was increased (Fig. 3a-b). In terms of response accuracy, the median of average response accuracy per participant at 1xJND was 62.54% (IQR = 43.57% − 67.97%), which significantly increased to 91.09% (IQR = 88.86% − 94.83%) at 2xJND shift intensity. Medians of average response accuracy further increased at the 3xJND and 4xJND levels to 96.35% (IQR = 93.84% − 97.11%) and 97.04% (IQR = 93.49% − 98.78%), respectively. A similar effect was observed in the case of reaction times for correct responses, which became faster with increasing shifts (Fig. 3c): median correct response times at 1xJND level were 1.44 s (IQR = 1.25–1.59 s). Each decrease in the shift reduced median response times, with 4xJND being the lowest at 0.69 s (IQR = 0.64–0.78 s), speeding up response times from 1x to 4xJND by almost 750 ms. These results imply that providing larger shifts in electrotactile stimulation improves discriminability and results in faster and more accurate perception.

**Fig. 4:**
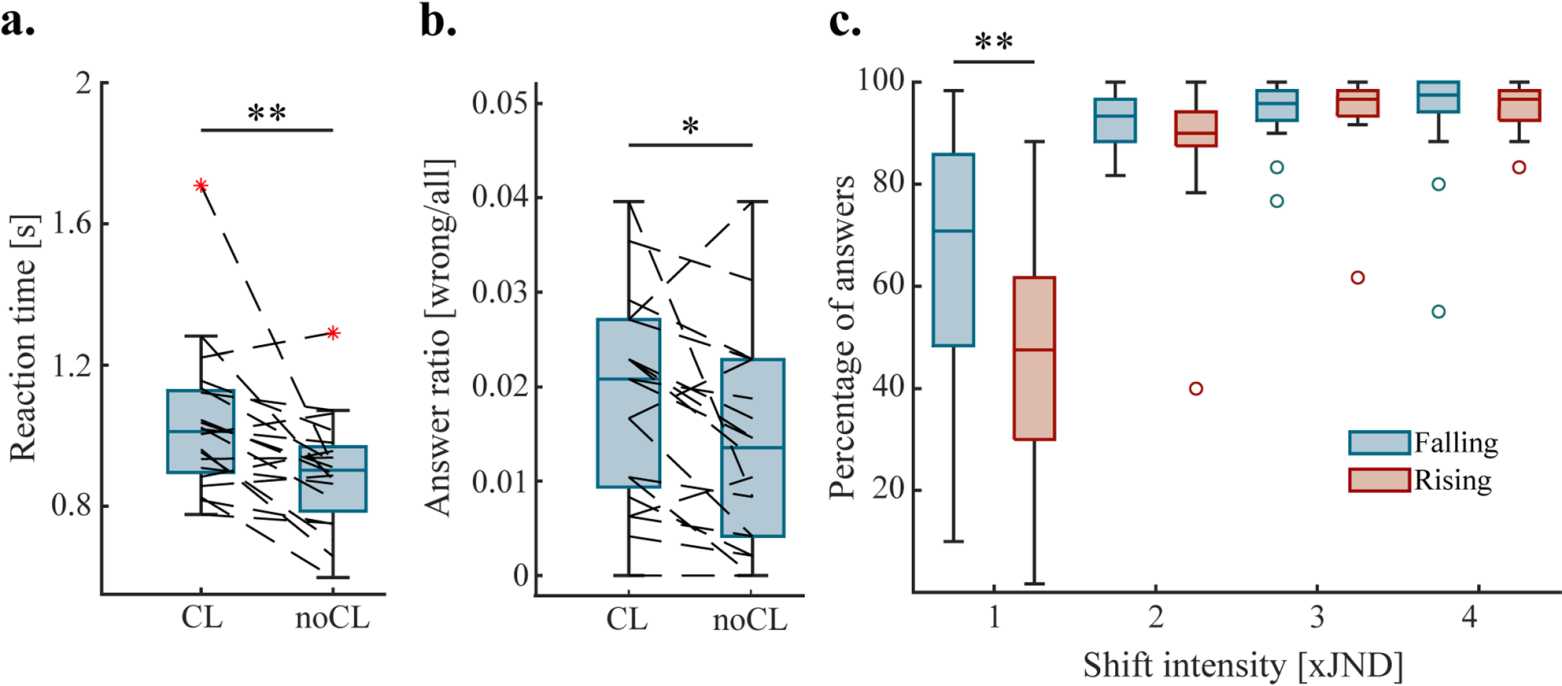
Effects of cognitive load (CL) and shift direction on reaction times and response accuracy; **a** Added cognitive load results in slower average reaction times; response accuracies do not change significantly. **b** Added cognitive load results in more incorrect responses; missed responses are not included and are unaffected (one outlier not pictured) **c** There are less correct responses on average for rising shift than falling shift for the smallest shift intensity; there is no significant difference at higher shift intensities

Performing the task with simultaneous cognitive load led to significantly slower reaction times (*p* < 0.001, Fig. 4a), with a median difference of 175ms. While the average number of accurate responses were not affected (*p* = 0.42), there was a significantly higher number of inaccurate responses in the cognitive load condition, not counting missed responses (*p* < 0.05, Fig. 4b). After blocks with the cognitive load condition, participants were asked to answer questions regarding the audiobook in the form of a MCQ test. Participants on average scored 79% ± 20.51% on the MCQ test, indicating that participants performed well on the secondary task despite their divided focus. In the case of shift direction (rising/falling intensity), the ANOVA revealed an interaction effect with shift intensity regarding response accuracies (*p* < 0.001). Further analysis using a Wilcoxon signed rank test revealed that a significant difference was only present for the lowest shift intensity (*p* < 0.01, Fig. 4c), making rising shifts harder to determine correctly. Explicit instructions regarding focus on response speed or response accuracy did not have a significant effect on either reaction times or response accuracies (*p* = 0.11 and *p* = 0.25, respectively). Further Bayes factor analysis confirmed these results, revealing BF_10_ = 0.23 and BF_10_ = 0.23 for reaction times and response accuracies, meaning moderate evidence in favor of the null hypothesis.

A Wilcoxon signed-rank test has been performed between the per-participant average of onset and terminal trials for both response times and response accuracies, which confirmed no significant effect in both cases (*p* = 0.39 and *p* = 0.88 respectively). Similarly, the per-participant average of early and late trials were compared for both response times and response accuracies, which confirmed no significant effect in both cases (*p* = 0.23 and *p* = 0.84 respectively).

## Discussion

Using a prosthetic device effectively requires its incorporation into the sensorimotor system. If supplementary feedback is added to the prosthetic device, its parameters need to be designed to provide fast and distinguishable information which the sensorimotor system can exploit. In this study, we showed that (1) bigger steps during shifting electrotactile stimulation lead to faster and more accurate responses and (2) the presence of cognitive load only increases response times to shifting electrotactile stimulation, but does not affect the number of correct responses. Furthermore, we have confirmed previous findings which demonstrate that reaction times to electrotactile stimuli are faster than those to visual stimuli. These results reinforce the notion that providing bigger steps during electrotactile stimulation lessen the effect of the SAT and make the perception of shifting stimuli easier. Therefore, providing electrotactile stimulation in bigger steps could improve closed-loop control of prosthetics by making stimuli faster and less cognitively demanding to respond to.

Current research efforts focusing on providing supplementary feedback for upper-limb prosthetics aim to improve control via encoding tactile or proprioceptive events [32–34]. In order to improve control, we have to ensure the feedback is properly integrated into the sensorimotor control loop [12]. Proper integration can be facilitated by providing feedback through a fast modality. While the advantage in reaction times for the tactile modality is promising, it is important to remember that sensory stimuli carry varied information and the process of distinguishing them takes time as well [17, 18]. In order to avoid delays and inaccuracies, the changes in sensory feedback need not only to be swiftly noticeable, but easily discernible as well. Our results show that introducing bigger steps in electrotactile stimulation intensity leads to positive effects for both response times and response accuracy. The effect of step size on the response times is interesting in the context of previous research, which reported that modulation of perceived intensity in small increments (1xJND) during a closed-loop tracking task was characterized by a noticeable control delay [49, 50]. Our results suggest that while small increments enable a large number of levels of stimulation within the DR, they also increase the time it takes to accurately process the sensation of shifted stimulation intensity.

The difference observed between reaction times in Experiment 1 and response times in Experiment 2 for electrotactile stimulation can be explained by the Drift Diffusion Model (DDM) [51]. According to the DDM, response times are the summation of detection time, the time it takes to decide on a response and the execution time of the motor response. Due to the same stimulation modality and the highly similar motor response (button press), we can assume detection time and motor response time for electrotactile stimulation to be nearly identical in both experiments. Thus, we hypothesize that the response time improvements we see at bigger shift intensities in Experiment 2 are the result of a reduction in the decision time due to an increased rate of evidence accumulation—corresponding to the drift rate parameter of the DDM—driven by the increase in sensory evidence strength.

While providing electrotactile stimulation using bigger steps leads to faster and more accurate response times, this approach reduces the number of possible steps in the DR, making continuously changing sensory information (e.g., grip force or limb aperture) more discretized. The aim of these experiments was to determine how we could optimize the pulse width for speed and accuracy. However, in prosthetic implementations, a trade-off will have to be made between the speed/accuracy and amount of feedback steps that are required to convey the necessary information. From this paper, we show that using bigger steps leads to faster and more accurate responses. As such, if future applications struggle with either delayed control [49, 50] or low accuracy, the introduction of bigger shifts in intensity should be considered. According to our thresholding measurements at the beginning of every experiment the average dynamic range can be divided into 3 or 4 levels of discrete and easily distinguishable intensities. In this setup, the 1 st level corresponds to the reference stimulus at 25% of the DR and the following 2 or 3 levels correspond to 4x and 3xJND steps, respectively. This hypothetically enables the encoding of 3 or 4 distinct tactile or proprioceptive events, depending on the shift intensity chosen, without going above the PT. While 3-4xJND might not be possible for every application, our results suggest that changing from steps of 1xJND to 2xJND will already have a big impact on both the speed and accuracy of response.

Prosthesis users experience heavy cognitive load arising from several sources, such as overreliance on the visual system [52] and the undergoing reorganization of the sensorimotor system [53]. Additionally, everyday situations require users to manipulate their device while performing a simultaneous task (e.g. holding a conversation while using the prosthesis) which further increases the amount of cognitive load. To simulate these circumstances, we introduced a cognitive load condition into the experiment. While our results imply that presence of cognitive load has no effect on the number of accurate responses, it is important to note that it results in a significant increase in response times and incorrect answers. The increase in response times may lead to control delays in a closed-loop control task, which is in line with existing research which has shown that presence of cognitive load slowed down movement during a closed-loop dual task for both invasive and non-invasive feedback [46]. However, a different study found no impairment in control performance in the presence of cognitive load [54]. This discrepancy could be the result of a difference in task nature, as the “box and blocks” task requires less rapid response to incoming stimuli compared to our task or the walking task.

During movement, choosing the appropriate motor command is dependent on incoming stimuli, which are subject to continuous change; this often results in us having to alter or adapt our motor commands as well [18]. When presented with a stimulus which requires a change in our motor command, we have to make a decision on how to alter our movement. This decision, ideally, is both fast and accurate in assessment of the change necessary; in reality, the speed-accuracy tradeoff in most situations prevents us from fulfilling both conditions at the same time. One of the most common ways to manipulate the SAT is through verbal instructions, as in explicitly instructing the participant whether to focus on speed or accuracy in their response [55–57]. While we implemented verbal instructions in our second experiment, we have failed to see significant differences in this condition regarding both response times and response accuracies, which could be attributed to the qualitative nature of verbal instructions in SAT tasks [20]. Another possible explanation for the similar performance in the ’speed’ and ’accuracy’ conditions is related to task difficulty: harder tasks tend to have slower response times and reduced accuracy, while in easier tasks response times are faster and response accuracies increase [58]. Although our results show low response accuracies and high response times in the 1xJND condition, both of these metrics improve significantly for the 2xJND condition. While we see further improvements regarding response times in the 3x and 4xJND conditions, average response accuracies already reach near 100% average accuracy for the 3xJND condition with a non-significant difference in the 4xJND conditions. A post-hoc Wilcoxon signed-rank test confirmed that the large number of missed responses at 1xJND are not related to the presence of cognitive load (*p* = 0.26). We hypothesize that the large number of missed responses arise from the fundamental definition of the just noticeable difference. In the context of alternative force choice tests (the method we used to determine JND), it has been established that the JND is the intensity level of stimulation the observer responds correctly to 75% of the time [59]. If we were to illustrate the distribution of response times and response accuracies via a psychometric curve, 1xJND would most likely represent the middle section (where we would expect to see the JND value by definition) [59, 60]. However, 2xJND already shows signs of the opposite tail end; this implies that to accurately establish a discrimination threshold - where we would see a difference in the verbal instruction condition -, as well as a psychometric curve, we would need to introduce additional shift intensities below 1xJND and between 1xJND and 2xJND [61, 62].

During use of a prosthetic device, the user heavily relies on their vision in order to gain feedback from the device. While vision provides reliable feedback, it lacks the advantage in speed the tactile modality possesses [21, 22, 24]. Together with the increase in cognitive load, this insufficiency in the speed of the visual modality introduces further delay into the sensorimotor control loop, further destabilizing the system. For this reason, supplementary feedback should be provided through a modality that is ideally faster than the visual modality. Although it has been shown before that tactile stimuli are generally registered faster than visual stimuli [63], this has not yet been confirmed for electrotactile stimulation. In order to test this, we performed a reaction time test where we compared average reaction times for both visual and electrotactile stimulation. Our results showed that average response times to electrotactile stimulation were ~ 50ms faster than visual stimuli, which confirmed our hypothesis. Moreover, this difference was already observed in the averages of the fastest 10% of participants’ reaction times between visual and electrotactile stimulation. The difference between reaction times already appearing in the leading edge of the reaction time distribution suggests that the advantage of electrotactile stimulation is due to the faster encoding of evidence rather than necessarily a change in the evidence accumulation process itself. Shifts in the leading edge of RT distributions in perceptual decisions are best explained by a difference in the non-decision time parameter of an evidence accumulation model, which encompasses both sensory encoding and motor processes [64]. The difference in sensory encoding time is thought to arise from differences in the mechanisms of the peripheral nervous system across sensory modalities, leading to variations in their associated reaction times [65]. The main limitation of our study was that testing was performed on able-bodied participants. However, the ability to distinguish between different intensities of electrical stimulation during a sensorimotor task were shown to be similar between able-bodied people and people living with limb-difference [36]. It is important to note that tasks included in this research were feed-forward in nature. While it is true that closed-loop tasks more closely reflect the control of a prosthetic device, these studies were necessary to determine the delays associated with different feedback modalities and their stimulation parameters. This study allowed us to identify important stimulation parameters for future testing in closed-loop control.

Our experiments employed adhesive 5 × 5 PALS electrodes placed over the flexor carpi radialis region of the non-dominant forearm. This configuration was selected to maximize participant comfort during extended sessions. While using electrodes of this size makes integration with prosthesis sockets or linings difficult, our goal in this study was to investigate perceptual and decision-making mechanisms under controlled and comfortable conditions, rather than to emulate a final prosthetic interface. Opinions regarding the effect of electrode surface area on perceived intensity are varied. Based on previous studies, we understand that electrode area impacts perception threshold [66, 67], but not discomfort thresholds [66]. We argue that while electrode size might shift sensation thresholds and dynamic ranges, it should not have a direct effect on the results and implications of this study.

Another limitation of this study was that it did not directly involve prosthesis use or myoelectric control, and stimulation was applied contralaterally to the active limb. This design choice was intentional, as it enabled the controlled investigation of perceptual and decision-related responses to electrotactile stimulus shift prior to implementation in a closed-loop prosthetic context. It should be noted that a methodological difference was present between the two experiments, namely the use of monophasic pulses in Experiment 1 and biphasic pulses in Experiment 2. This discrepancy arose from a change of the stimulation hardware: the initial microcontroller-based setup supported only monophasic output, while the subsequent system permitted generation of biphasic signals. We chose to change the pulse type in the second experiment as the experiment was noticeably longer, and biphasic stimulation is generally favored for its improved charge balance and reduced risk of skin irritation or discomfort during extended use [68, 69]. However, the relatively low stimulation intensities applied in Experiment 1 render it unlikely that this variation considerably affected perceptual outcomes or task performance.

In terms of both response times and response accuracies for Experiment 2, post-hoc analysis showed no significant effect that could be attributed to sensory habituation. While this cannot provide direct information on habituation in future prosthetic implementation, the results indicate that it most likely did not affect participant performance in this study. Additionally, we have shown that bigger shift intensities result in improved response times and response accuracies for a single stimulus shift compared to an established reference intensity. During closed-loop prosthetic control, however, stimulation can change on a much faster time scale. While we hypothesize that the benefits of faster and more accurate responses carry over to multiple stimulus shifts happening in a small period of time, this should be confirmed in future experiments. Lastly, we used an ANOVA to determine possible interactions and the influence of parameters at group level. However, not all subsets of the data were normally distributed. This decision was made due to the lack of suitable alternatives to ANOVA for non-normally distributed datasets. All subsequent comparisons between subsets were performed via methods suited for non-normally distributed data.

## Conclusions

In this study, we explored how to optimize electrotactile stimulation for prosthetic feedback to be integrated into the sensorimotor system. We have shown that bigger steps during shifting electrotactile stimulation lead to faster and more accurate responses. Our results also show that presence of cognitive load only increases response times to shifting electrotactile stimulation, but does not worsen response accuracy. Therefore, providing electrotactile stimulation in bigger steps could improve closed-loop control of prosthetics by making electrotactile stimuli easier and less cognitively demanding to respond to.

## Data Availability

The datasets used and analyzed during the current study are available from the corresponding author on reasonable request.
